# Water, Collagen, and Lipid Content in the Human Skin and Muscles Assessed with Near‐Infrared Diffuse Reflectance Spectroscopy and Multi‐Spectral Optoacoustic Tomography

**DOI:** 10.1002/advs.202505619

**Published:** 2025-08-13

**Authors:** Denis Davydov, Alexey Kurnikov, Pavel Subochev, Gleb Budylin, Nikolay Fadeev, Ivan Filippov, Natalia Mokrysheva, Liliya Urusova, Daniel Razansky, Evgeny Shirshin

**Affiliations:** ^1^ Laboratory of Clinical Biophotonics Institute for Regenerative Medicine I.M. Sechenov First Moscow State Medical University Moscow 119048 Russia; ^2^ Medical research and Educational Institute Lomonosov Moscow State University Moscow 119991 Russia; ^3^ Laboratory of ultrasound and optoacoustic diagnostics Division of radiophysics methods in medicine A.V. Gaponov‐Grekhov Institute of Applied Physics RAS Nizhny Novgorod 603950 Russia; ^4^ Faculty of Рhysics M.V. Lomonosov Moscow State University Moscow 119991 Russia; ^5^ Laboratory of Endocrine Biophotonics Endocrinology Research Center Moscow 117292 Russia; ^6^ Institute for Biomedical Engineering and Institute of Pharmacology and Toxicology Faculty of Medicine University of Zurich Zurich 8057 Switzerland; ^7^ Institute for Biomedical Engineering Department of Information Technology and Electrical Engineering ETH Zurich Zurich 8093 Switzerland

**Keywords:** collagen, diffuse reflectance spectroscopy, lipids, multi‐spectral optoacoustic tomography, muscles

## Abstract

Infrared spectroscopy can quantify individual body components such as lipids, water, and proteins, but extending it to a comprehensive assessment of overall body composition is hampered by high variability and optical heterogeneity of biological tissues. Here, a theoretical and experimental strategy merging multi‐spectral optoacoustic tomography (MSOT) and diffuse reflectance spectroscopy (DRS) is introduced to characterize skin and subcutaneous tissue composition in the near‐infrared range. Water, lipids, and collagen exhibit distinct absorption peaks, with lipids demonstrating significantly higher absorption than collagen at comparable mass concentrations. Diminished lipid absorption in subjects with thin hypodermis allows the DRS method to detect distinct collagen band at 910 nm, whose magnitude correlates with the muscle mass, as confirmed by bioimpedance analysis. Conversely, strong lipid peak at 930 nm in subjects with pronounced hypodermis overshadows collagen signals by an order of magnitude, making DRS characterization insufficient. MSOT overcomes this limitation by offering high‐resolution depth‐resolved 3D imaging to accurately delineate the dermis, hypodermis, and muscle layers in vivo and quantify each chromophore's contribution individually. The findings demonstrate the complementary capabilities of MSOT and DRS for molecularly specific, noninvasive body composition analysis, potentially enhancing diagnostic approaches for a number of conditions, such as obesity and sarcopenia.

## Graphical abstract

Authors present a novel approach combining multispectral optoacoustic tomography and diffuse reflectance spectroscopy for noninvasive assessment of body composition, including water, lipid, and collagen in skin and muscles. Its high molecular and spatial specificity offers improved diagnostic potential for conditions like obesity and sarcopenia.

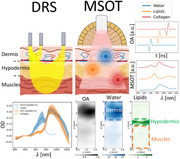

## 1 Introduction

Development and refinement of non‐invasive methods for body composition analysis is important for ensuring convenient, accurate, and safe diagnosis and monitoring of diseases.^[^ ^1^ ^]^ The existing methods for body composition analysis exhibit high variability in terms of invasiveness, cost, accuracy and complexity. Among the non‐invasive, accurate, yet complex and expensive clinical methods, are the magnetic resonance imaging and X‐Ray computer tomography (CT) modalities. Although being considered as a gold standard in the body composition analysis, the low accessibility, use of ionizing radiation, and relatively long analysis time limit their everyday application.^[^ ^2^ ^]^

Unlike clinically established imaging methods, infrared spectroscopy offers enhanced molecular specificity and sensitivity by probing the fundamental vibrational modes of proteins, lipids, and water. However, the strong absorption of water in the mid‐infrared (mid‐IR) region prevents Fourier transform infrared (FT‐IR) spectroscopy^[^ ^3^ ^]^ and terahertz (THz) spectroscopy^[^ ^4^ ^]^ from quantifying water, lipids and protein content in deep skin layers. Raman spectroscopy can differentiate between water and lipids due to distinct differences in their vibrational spectra. Yet, its penetration depth is limited to ≈100 µm due to the high autofluorescence background of the skin. Consequently, diffuse reflectance spectroscopy (DRS) and multispectral optoacoustic tomography (MSOT) in the near‐infrared (NIR) window are currently regarded as the most promising approaches for determining the molecular composition of the skin and subcutaneous tissue, as they enable interrogation depths of up to ≈1 cm. A comparative analysis of spectroscopy methods versus non‐optical methods is presented in **Table** **1**.

**Table 1:** Comparison of optical and non‐optical methods for tissue composition analysis.

Non‐Optical Methods				
Method	Contrast Mechanism	Measured Parameters	Limitations	Refs.
MRI	Proton relaxation (T1, T2, PD); chemical shift	Water and fat content; muscle distribution	High cost; long scan time; limited molecular specificity	[2, 5]
DEXA	Dual‐energy X‐ray attenuation	Bone density; fat and lean mass	Ionizing radiation; low resolution; limited molecular specificity	[5, 6]
Bioimpedance Analysis	Frequency‐dependent impedance	Total body water, fat and lean mass	Indirect measurements; no depth resolution	[7]
Ultrasound	Acoustic impedance and backscatter	Tissue thickness; muscle quantity and quality	Operator‐dependent; no molecular specificity	[8]
Myography	Mechanical muscle oscillations during contraction	Muscle activity and function	Surface muscle only; no biochemical information	[9]

Optical Methods				
Method	Contrast Mechanism	Measured Parameters	Limitations	Refs.
FT‐IR	Absorption of mid‐IR fundamental vibrational modes	Proteins, lipids, water (in thin samples)	Strong water absorption; penetration depth <10 µm	[3]
THz Spectroscopy	Terahertz absorption and reflection	Water content; hydration status	Penetration depth <10 µm; high sensitivity to water	[4]
Confocal Raman Spectroscopy	Inelastic light scattering (vibrational modes)	Lipids, water, proteins	Penetration depth ≈100 µm; high autofluorescence background	[10, 11]
Second Harmonic Generation	Nonlinear signal from non‐centrosymmetric molecules	Collagen, myosin	No signal from lipids/water; organized proteins only; shallow depth	[12, 13]
DRS	Wavelength‐dependent scattering and absorption	Water, lipids, hemoglobin, collagen	Low spatial resolution; requires calibration for quantification	[13, 14, 15, 16]
MSOT	Light absorption, ultrasonic wave generation	Water, lipids, hemoglobin, collagen	High cost; resolution degrades with depth	[17]

**Figure 1:**
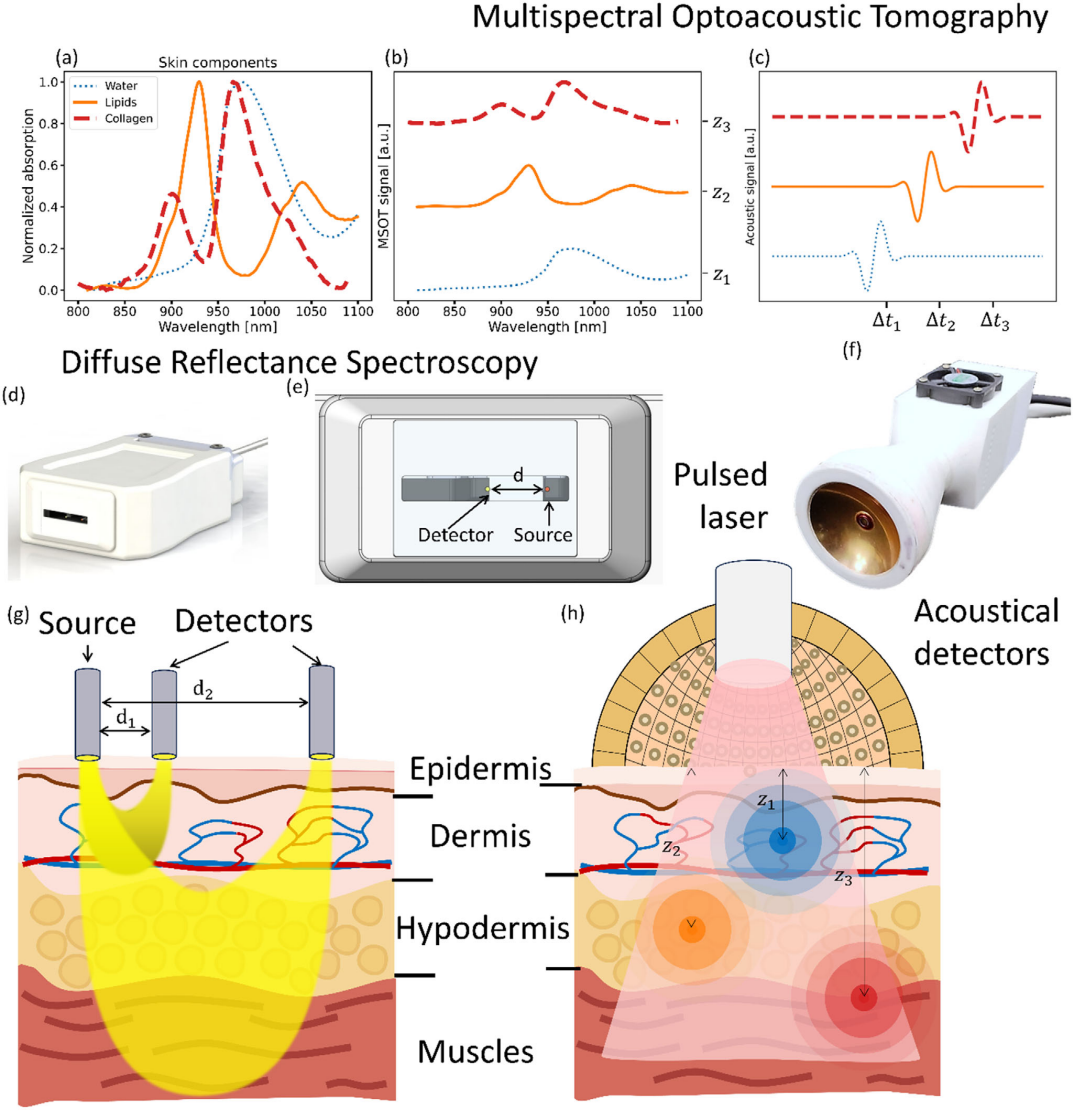
Comparison of Multispectral Optoacoustic Tomography (MSOT) and Diffuse Reflectance Spectroscopy (DRS) for imaging skin structures. a) Normalized absorption spectra of major skin chromophores: water, lipids, and collagen. b) Schematic of MSOT signals acquired at different tissue depths (z_1_, z_2_, z_3_) across a range of wavelengths. c) Schematic of time‐resolved optoacoustic waveforms corresponding to signals originating from different depths, i.e., different propagation delays of the acoustic wave following emission of the laser pulse (Δt_1_, Δt_2_, Δt_3_). d,e) Representative DRS probe and f) spherical matrix array transducer used in MSOT (right). g) Schematic representation of DRS demonstrating light delivery and detection in superficial skin layers (epidermis, dermis, and hypodermis). h) MSOT schematic illustrating laser‐induced ultrasound generation and detection, enabling depth‐resolved imaging of subsurface structures.

Both DRS and MSOT methods can operate at centimeter‐scale imaging depth,^[^ ^18^ ^]^ sufficient for analyzing the skin and subcutaneous layers with functional and molecular specificity (**Figure** **1**a–c). DRS is based on measuring light undergoing multiple scattering events and being partially absorbed within the tissue, which is typically done with a fiber‐optic probe featuring separate source and detector fibers (Figure 1d,e). Spatial resolution is achieved by varying the distance between the source and detector fibers (Figure 1g). This allows assessing concentrations of various tissue components, such as water,^[^ ^16^, ^19^, ^20^, ^21^, ^22^ ^]^ lipids,^[^ ^15^, ^23^ ^]^ hemoglobin,^[^ ^24^, ^25^ ^]^ and collagen.^[^ ^26^, ^27^, ^28^ ^]^ The MSOT method detects instead ultrasound signals generated through light absorption by tissue chromophores (Figure 1a,b). It uses a wavelength‐tunable nanosecond pulsed laser source and multi‐element ultrasound transducer (Figure 1f), which tomographically records the generated optoacoustic responses (Figure 1c). As a result, MSOT allows obtaining 3D images with high spatial resolution and molecular contrast (Figure 1b,h).^[^ ^29^ ^]^ It has been proven to be effective for characterizing vascular morphological parameters,^[^ ^30^, ^31^, ^32^ ^]^ assessing blood oxygenation in tissues,^[^ ^33^ ^]^ tracking contrast agent perfusion and biodistribution.^[^ ^34^ ^]^

The use of MSOT for determining tissue composition and morphology in vivo beyond the vasculature assessment is limited by its low anatomical contrast. Therefore, MSOT data are often combined with ultrasound images of the interrogated tissue to enhance the morphological contrast.^[^ ^17^, ^35^, ^36^, ^37^ ^]^ However, isolating individual molecular components in different tissue layers remains a daunting task. The highly heterogenous distribution of optical absorption and scattering in living mammalian tissues requires detailed modeling of light propagation, which is further challenged by the so‐called spectral coloring effects^[^ ^38^ ^]^ and significant discrepancies in the reported spectral shapes of water, lipid, and collagen components.^[^ ^39^, ^40^, ^41^ ^]^

Although the combination of MSOT with other modalities has been actively investigated, the integration of MSOT with DRS remains insufficiently explored, as noted in a recent review,^[^ ^42^ ^]^ despite several early studies demonstrating such combinations using optical phantoms,^[^ ^43^, ^44^ ^]^ and *ex vivo* tissues. Subsequent work extended these studies to *ex vivo* biological specimens, including tumor tissue^[^ ^45^ ^]^ and muscle tissue samples.^[^ ^46^ ^]^ More recently, our group applied this approach in vivo for tumor studies,^[^ ^47^, ^48^ ^]^ though only with single‐wavelength imaging.

The present study demonstrates the application of a multi‐modal imaging approach combining DRS and MSOT for non‐invasive body composition analysis. To correlate the optical signals with specific tissue constituents, we first validated the method on skin mimicking phantoms and then performed in vivo measurements on volunteers spanning a wide range of anthropometric characteristics. Comparison of the fundamental vibrational bands obtained by Raman spectroscopy with the overtone absorption bands recorded in the near infrared allowed for a more reliable identification of the spectral signatures of water, lipids and collagen in the skin and subcutaneous layers. The quantitative results obtained from the optical techniques were compared with multispectral bioimpedance analysis (mBIA), which served as an independent, clinically accepted reference method.

## 2 Results

### 2.1 Relationship Between Vibrational Spectra of Collagen, Lipid, and Water and their Optical Spectra in the NIR

**Figure 2:**
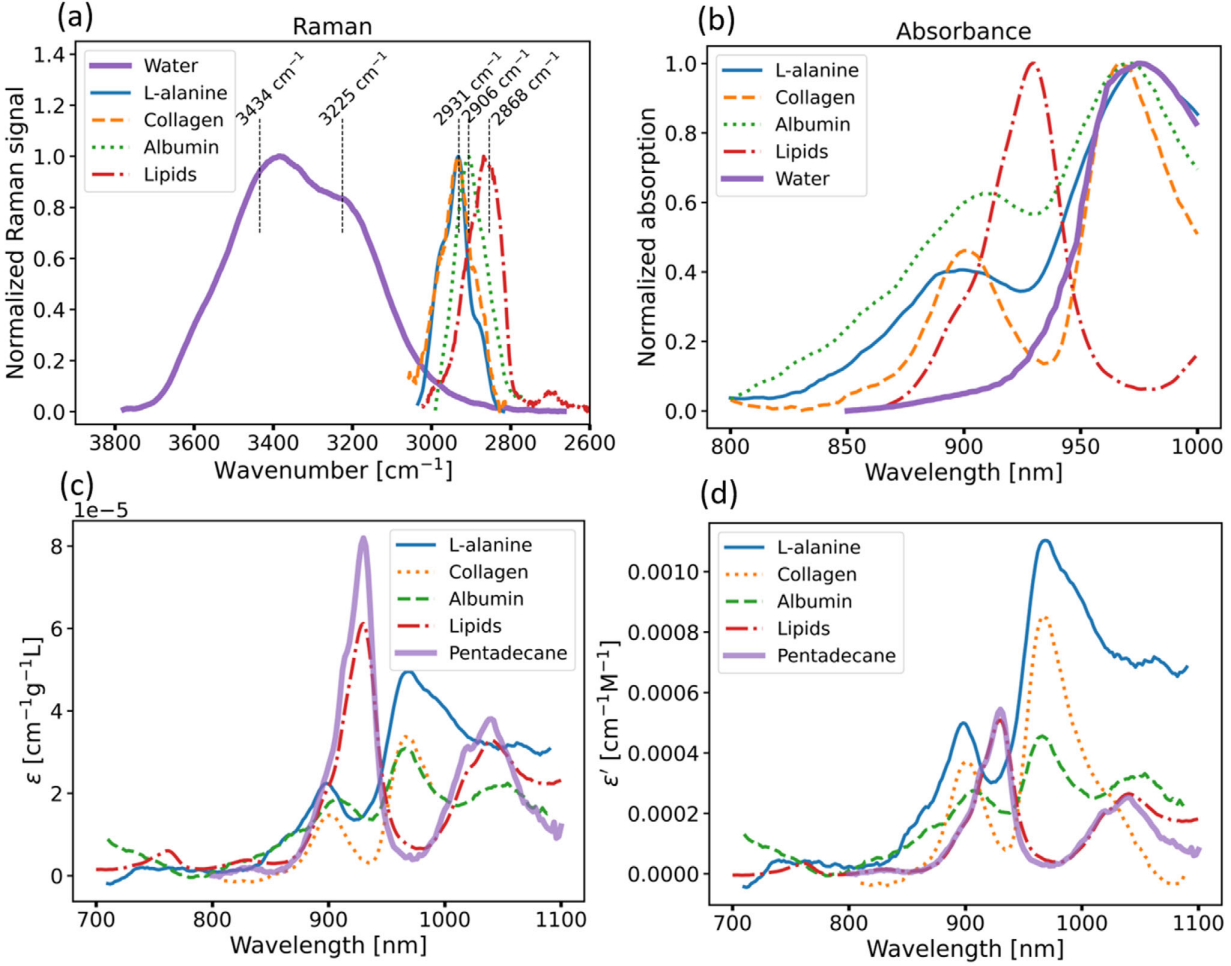
a) The Raman spectra of L‐alanine, albumin, water, and lipids. b) The absorption spectra of L‐alanine, albumin, water, lipids, and collagen. A third‐degree polynomial intersecting the spectrum in the ranges of 700–800 nm and 1050–1100 nm was subtracted for clarity in determining the position of the collagen absorption maximum. c) Absorption spectra of L‐alanine, albumin, lipids, pentadecane, and collagen, normalized by mass concentration. d) Specific molar extinction spectra of L‐alanine, albumin, lipids, pentadecane, and collagen, normalized by the number of C─H bonds.

The NIR absorbance of water, collagen, and lipid can be interpreted by considering their vibrational properties. For this, Raman and absorption spectra of these substances are presented in **Figure** **2**a,b. The maximum at 2854 cm^−^1 for C─H vibrations in lipids corresponds to an absorption maximum at 930 nm for the C─H vibrational overtones. For l‐alanine and albumin, the characteristic values are 2931 and 2906 cm^−^1 corresponding to 918 and 926 nm absorption bands, respectively. The characteristic absorption maximum for collagen is observed at 910 nm. Thus, different shapes of vibrational spectra lead to different shapes of the overtone absorption bands in the NIR range, making the spectral unmixing of chromophores possible.

Unlike for UV–vis spectra where electronic transitions in a few pi‐conjugated systems within the molecular structure are responsible for the optical spectra formation, multiple oscillators per molecule contribute to its absorption properties in the NIR range. Specifically, Figure 2c presents the absorption spectra of the model collagen‐ and lipid‐like chromophores normalized to their mass concentration in a sample. The amplitude of the C─H absorption band for collagen (near the local maximum at 910 nm) is several times smaller than that for lipids (near the local maximum at 930 nm) at the same mass concentration. **Table** **2** lists the molecular masses and estimates of the number of C─H bonds and extinction coefficients for the investigated molecules based on their structural formulas. For l‐alanine and pentadecane these values can be accurately calculated, while for other compounds the estimates were obtained from literature. The molar extinction spectra, normalized to the number of C─H bonds, are shown in Figure 2d. The amplitude of the NIR absorption, when normalized to the number of C─H bonds, is comparable for collagen and lipid. However, at the same mass concentration the absorption of collagen can be expected to be several times lower compared to lipids, thus suggesting lower sensitivity of both DRS and MSOT in collagen assessment.

**Table 2:** Structural formulas of collagen and lipid molecules and other model systems, their calculated molar masses (M) and the number of C─H bonds per molecule N(C‐H), absorption spectrum maximum near the C─H absorption band $\left(\varepsilon_{max}\right)$, normalized to mass concentration, and the maximum molar extinction spectrum near the C─H absorption band, normalized to the number of C─H bonds ($\varepsilon_{max}^{\prime }$). Calculations were performed using an open database.^[^ ^49^ ^]^

Sample	Structural formula	M [g mol^−1^]	N(C‐H)	$\varepsilon_{max}\;$[10^−5^ cm^−1^g^−1^L]	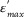 [cm^−1^M^−1^]
L‐alanine	C_3_H_7_NO_2_	89	4	2.2	0.005
Pentadecane	C_15_H_32_	212	32	6.1	0.004
Lipid	CH_3_(CH_2_)_n_COOH	870	100	8.2	0.004
Albumin	C_123_H_193_N_35_O_37_	66 500	3 600	1.8	0.004
Collagen	C_4_H_6_N_2_O_3_R_2_·(C_7_H_9_N_2_O_2_R)_n_	285 000	11 200	1.4	0.003

The presented spectral data indicate that three classes of components, namely, water, collagen, and lipid, can be potentially disentangled in the DRS and MSOT spectra measured from biological tissues, including the skin. While the peaks of lipids and water have previously been analyzed and used for diagnostic purposes, the absorption of collagen in the 910 nm region has not been assessed in detail, neither in the context of collagen quantification in the dermis nor in the context of muscle characterization.

### 2.2 Identification of the Skin Components In Vivo using DRS: Fat and Muscles

**Figure 3:**
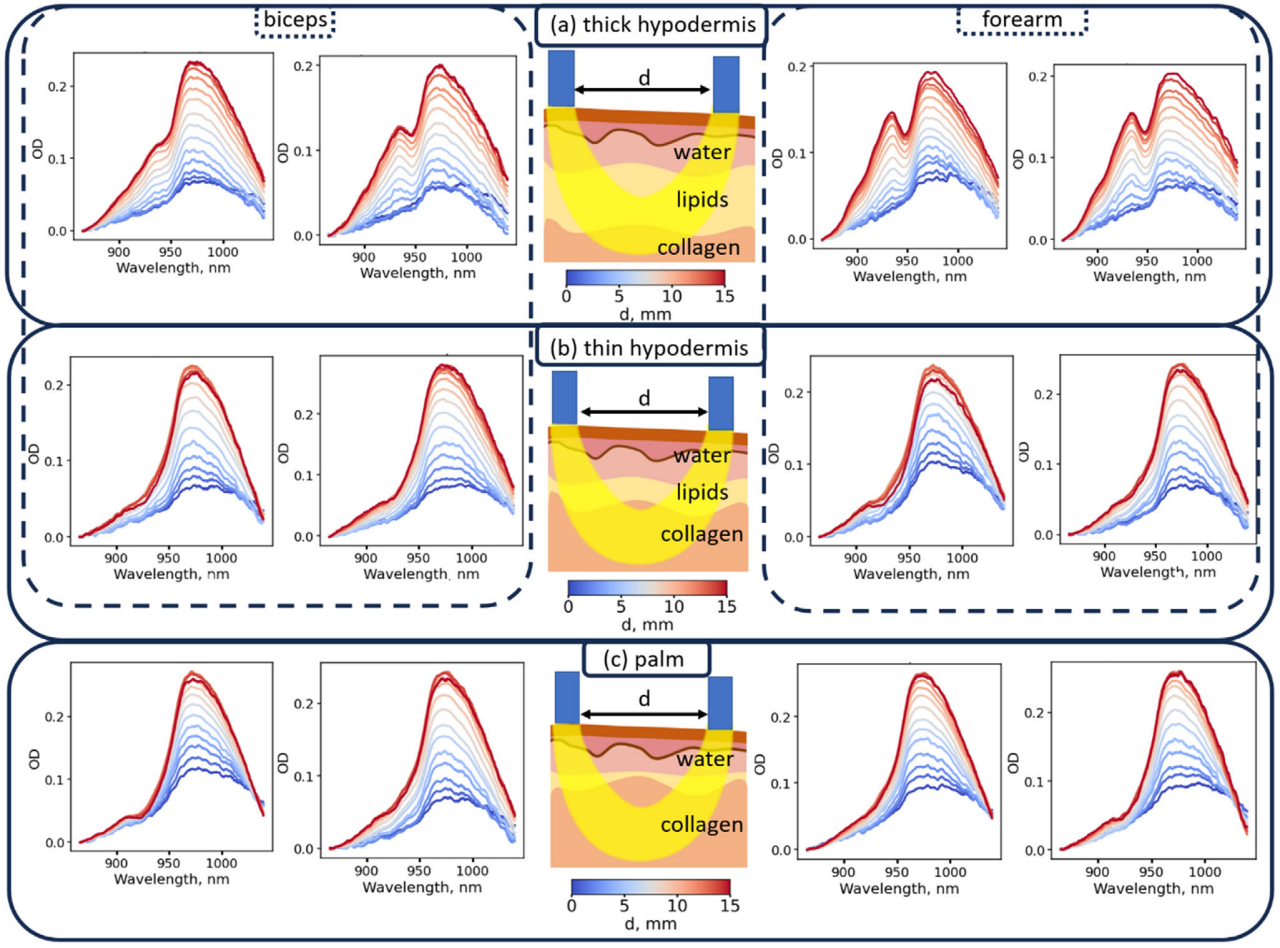
Diffuse reflectance spectra of volunteers measured from a) the bicep and forearm for a thick hypodermis, b) same for a thin hypodermis, and c) the palm. The gradient from blue to red corresponds to distances between the DRS fibers ranging from 0 to 15 mm.

Spatially‐resolved DRS was performed for various skin areas – lower forearm, biceps area, and palm – to assess the impact of collagen, water, and lipids. Our previous works,^[^ ^15^, ^50^ ^]^ as well as other independent studies,^[^ ^23^, ^51^ ^]^ have identified a positive correlation between the hypodermis thickness and the lipid absorption peak at ∼930 nm. For volunteers with well‐defined hypodermis on the forearm and biceps, we observed a characteristic 930 nm lipid absorption peak and 970 nm water absorption peak (**Figure** **3**a), while the collagen‐related peak was not pronounced. Upon increase of the source‐detector separation from 0 to 15 mm (Figure 3), ratio of the lipid to water absorption increases. This is attributed to the higher average photon propagation length and probing depth – the impact of lipids from the hypodermis increases with the effective detection depth.^[^ ^14^, ^15^ ^]^

Furthermore, the 930 nm absorption peak was absent in volunteers with minimal hypodermis thickness (Figure 3b). On the contrary, a characteristic collagen absorption peak at 910 nm for longer (>5 mm) separations between the source and detector was clearly observed. These observations indicate that the NIR DRS analysis of the skin can go beyond the water‐lipids paradigm by considering the muscle layer.

For all volunteers, we observed an evident collagen‐related absorption peak around 910 nm in the palm for long (>5 mm) separations between the source and detector (Figure 3c). No lipid absorption peak was observed in the palm skin area, which is consistent with ultrasound data and previous works.^[^ ^15^, ^50^ ^]^

Hence, the analysis of the DRS signal allows mapping the spatial distribution of molecular components in the skin. Previously, we demonstrated tracking of hydration changes in skin layers under various loads with spatially resolved DRS.^[^ ^22^ ^]^ It also allows for determining body fat content^[^ ^14^ ^]^ and the thickness of the dermis and hypodermis.^[^ ^15^ ^]^ In the current work, in addition to water and lipids, muscle layer analysis has successfully been conducted based on the characteristic collagen spectrum, which becomes detectable in areas with thin hypodermis.

In the case of a thin hypodermis, a characteristic increase in the Collagen Index to Lipid Index ratio is observed with increasing source‐detector separation. In contrast, for a thick hypodermis, this ratio remains constant as the source‐detector distance increases. This effect is attributed to the spectral overlap between collagen and lipid absorption bands, which prevents accurate estimation of collagen content under conditions of high lipid absorption. The results indicate that reliable collagen quantification using DRS data becomes unfeasible with source‐detector separation of 10 mm or less and Collagen Index/Lipid Index ratio below 0.5 (Figure S1, Supporting Information).

### 2.3 Comparison of Bioimpedance Analysis and DRS in Body Composition Analysis

**Figure 4:**
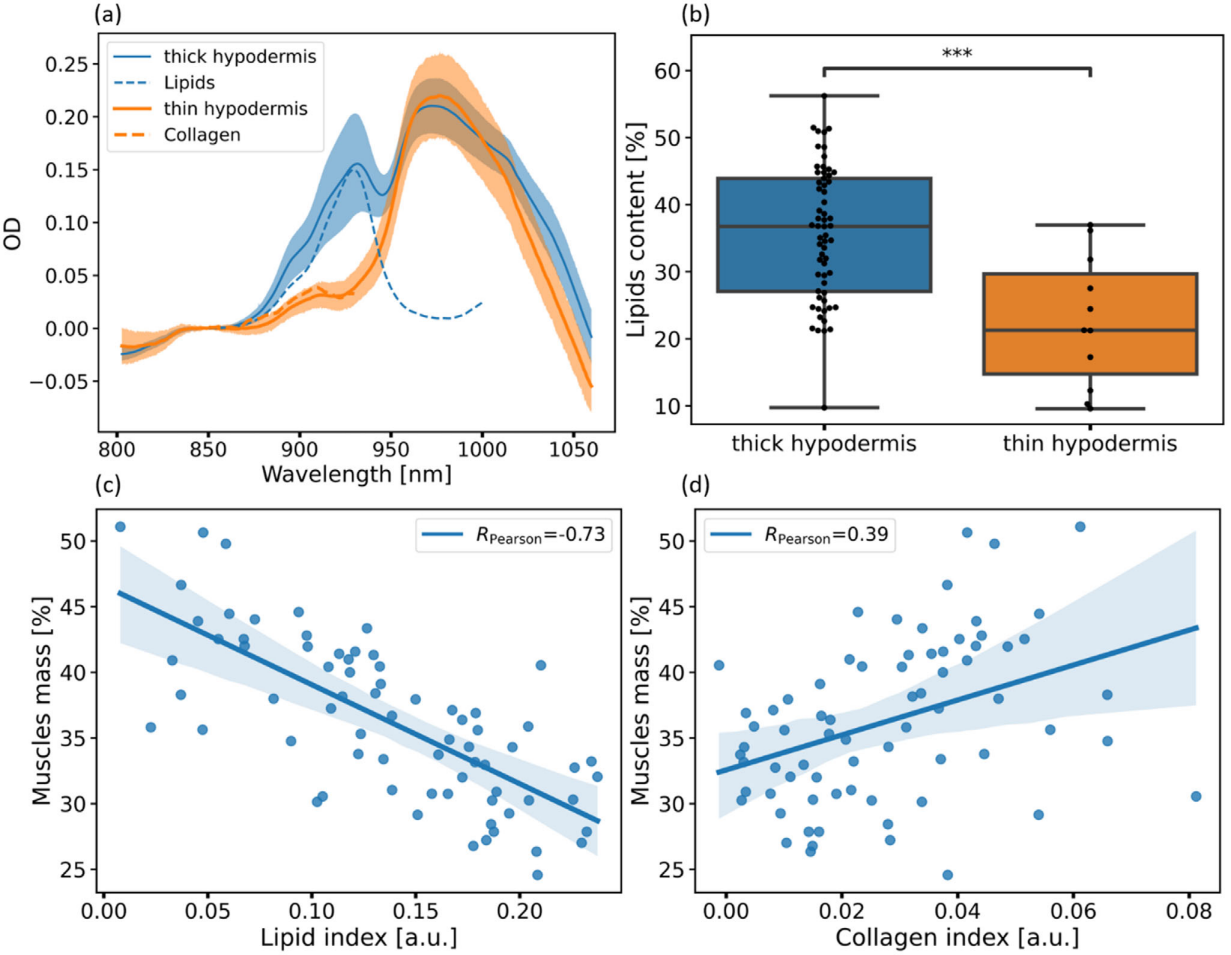
a) Optical density spectra for skin with thick and thin hypodermis over the wavelength range of 800–1050 nm, showing distinct spectral features between the two groups. For clarity, the lipid absorption spectrum is shown by the blue dashed line, and the collagen absorption spectrum is shown by the orange dashed line. b) Box plots of the percentage of fat content for volunteers with thick (n_1_ = 61) and thin hypodermis (n_2_ = 11) (independent‑samples *t*‑test, *p* = 1.3 10^−4^). c) The dependence of the lipid absorption amplitude plotted versus the muscle mass percentage provided by BIA (sample size n = 72, Pearson R = –0.73, *p* ≪ 10^−3^). d) The dependence of the collagen absorption amplitude plotted versus the muscle mass percentage provided by BIA (sample size n = 72, Pearson R = 0.39, *p* = 10^−3^).

The possibility to extract the collagen‐related peak in the DRS spectra as a proxy of the muscles content was further evaluated. For this, a set of volunteers measured by the mBIA was divided into two groups (**Figure** **4**a): 1) subjects with a thin hypodermis, for which a characteristic collagen (muscles) local absorption maximum was observed at ≈910 nm (orange curves), and 2) subjects with a thick hypodermis, exhibiting a lipid absorption peak at ∼930 nm (blue curves). There was a statistically significant difference in the percentage of fat between the designated subject groups (*p* = 1.3 10^−4^,t_statistic_ = 4, Figure 4b), which had no significant differences in age or body mass index (BMI) (Figure S2, Supporting Information). A correlation between the muscle mass percentage provided by BIA and the amplitude of the lipid absorption peak obtained at a 15 mm source‐detector separation was observed (Pearson R = –0.73, p ≪ 10^−3^; Figure 4c). Furthermore, a correlation was also detected between muscle mass and the magnitude of the collagen absorption peak ∼ 910 nm estimated from the DRS (Pearson R = 0.39, p = 10^−3^; Figure 4d). These findings further confirm the relationship between the 910 nm absorption peak in the DRS spectra, and the muscle layer beneath the skin.

Importantly, for the subjects with thick hypodermis, where the lipids peak at 930 nm could be observed, the collagen‐related peak was not detectable. We have previously elaborated on this observation: at similar mass concentration the absorbance of lipids is several folds higher compared to the dominant skin protein collagen due to much higher concentration of the C─H oscillators (NIR absorbers) per molecule (see Table 2). As a result, the presence of lipids masks the collagen absorbance in the DRS spectra as it measures the signal integrated along the whole diffuse photon propagation trajectory. Indeed, the collagen mass fraction both in the dermis and in muscles is about 10%, while the lipids mass fraction in the hypodermis exceeds 70%. Hence, the use of DRS for quantification of the muscles layer is limited for subjects with high BMI and pronounced hypodermis. To overcome this limitation, spatial gating must be used, i.e., the signal must be collected selectively below the hypodermis layer. Such spatial localization can be performed with MSOT, where the depth from which the signal is detected can be selected by time gating of the absorption‐generated acoustic wave. We thus explored instead the hypothesis that MSOT allows for accurate muscle assessment using the collagen absorption peak at 910 nm, even in the presence of pronounced hypodermis.

### 2.4 The Spectra of MSOT Signal in Muti‐Layered Tissue Phantoms

As the DRS measurements do not provide accurate information about spatial location or depth of the chromophores, we used MSOT in the same NIR spectral range of DRS, aiming at rendering spatially‐resolved 3D maps of chromophores including the skin layers. The experiments were first performed in multi‐layered optical phantoms. The detailed description of the fabricated phantoms and the rationale for their selection are provided in the Experimental Section.

**Figure 5:**
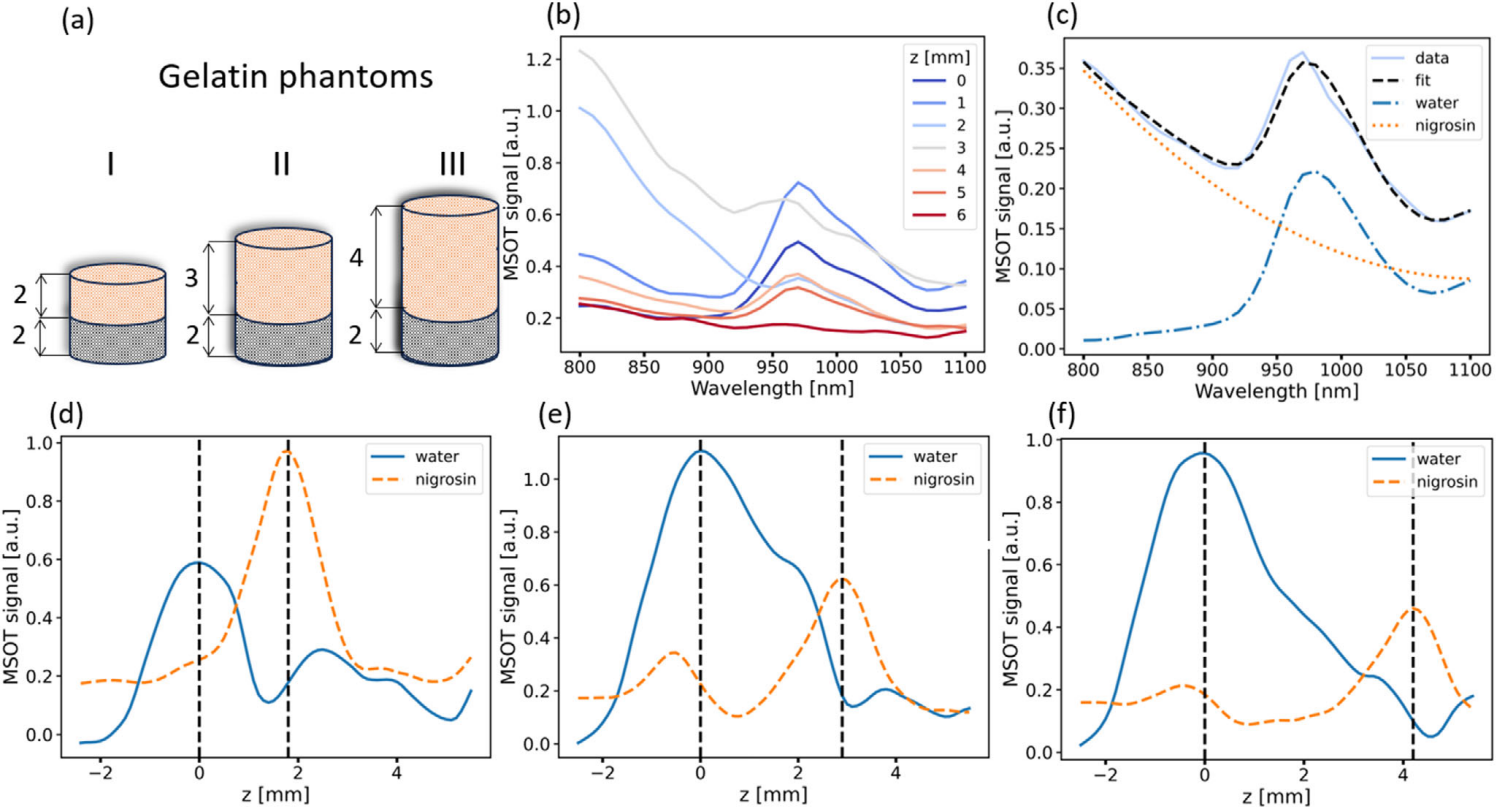
a) Schematic of layered phantoms with varying thickness of the first layer representing optical properties of the dermis. b) MSOT spectra averaged along the lateral coordinates for different depths along the z‐axis for phantom I. c) An example of fitting the MSOT spectrum to a sum of water and nigrosine absorption. d–f) Depth profiles of water and nigrosine components for gelatin phantoms with thickness of 2, 3, and 4 mm.

Unlike the DRS method, which measures the integrated reflected signal, MSOT can directly select the depth from which the signal is detected based on time of arrival of the generated optoacoustic responses. First, MSOT signal from two‐layered structures made of gelatin phantoms with different thickness was measured. The nigrosine dye was added to the second layer to make the two layers spectrally distinct, as in the case of skin layers (**Figure** **5**a). Since optical properties of the phantom remain constant along the lateral coordinates, the MSOT signal was averaged with the 1D depth profiles of the MSOT spectra analyzed (Figure 5b). To identify the components related to the concentration of water and nigrosine, spectral approximation was performed. The average approximation error remained below 5% (Figure 5c).

Figure 5d–f shows the amplitude dependencies of the spectral components on depth for gelatin phantoms. The upper phantom is characterized by high water absorption and the substrate is characterized by high nigrosine absorption. To reduce the influence of scattered acoustic signal on surface inhomogeneities of the phantoms, the detected signals were filtered up to 1.5 MHz, which has limited the effective spatial resolution and led to broadening of the maxima in the depth profile of the obtained components. The distances between absorption maxima along the z‐coordinate for each phantom were 1.9, 2.9, and 4.1 mm, respectively. These values correspond well to the thickness of the top layer, thus confirming that MSOT allows spatially localizing molecular components in skin‐mimicking structures. Although the phantom model is assumed to be homogeneous along the lateral dimensions, analysis of the spatially‐resolved MSOT signals at different lateral positions enables accounting for any potential inhomogeneities.

### 2.5 Identification of Skin and Subcutaneous Tissue Components in Humans Using MSOT

**Figure 6:**
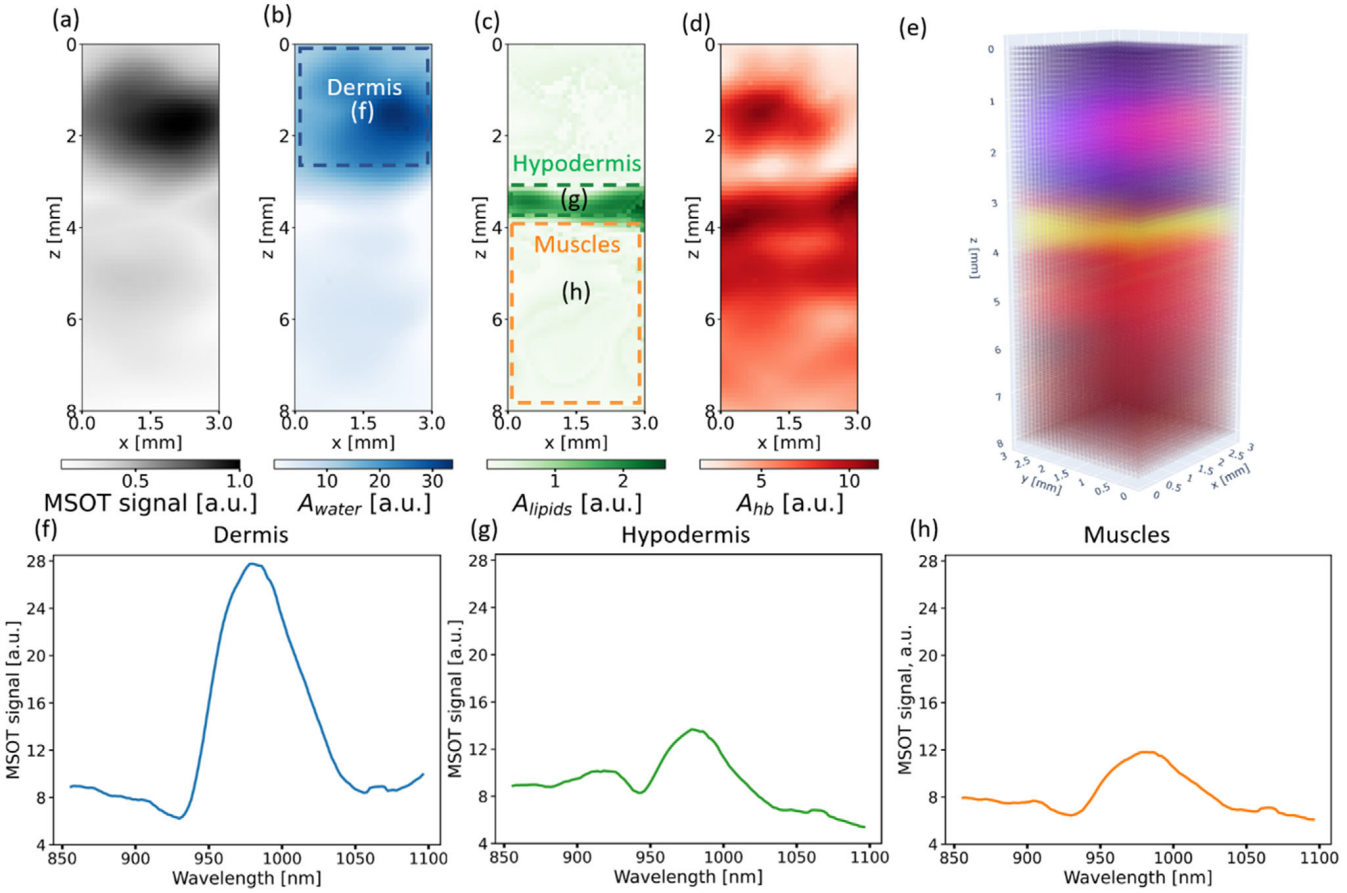
a) Projected 2D map of single‐wavelength MSOT signal. 2D map of spectrally unmixed water b), lipids c), and hemoglobin d) concentrations. e) 3D map of components associated with the absorption of water, lipids, and hemoglobin. Spatially‐averaged MSOT signal spectra for characteristic regions with the highest absorption of water f), lipid g), and collagen h).

In vivo skin measurements were subsequently performed with MSOT. **Figure** **6**a shows a characteristic single‐wavelength MSOT image projected along one of the lateral axes (i.e., averaged along the y axis). Spectral decomposition was then performed to extract the concentration maps of components associated with water (Figure 6b), lipids (Figure 6c), and hemoglobin (Figure 6d). Water absorption is localized in the upper part of the scan, corresponding to the dermis. Bright local areas with high total hemoglobin absorption, presumably corresponding to blood vessels, are observed in the projected 2D maps. One can also observe a region (layer) with high lipid absorption, which corresponds to hypodermis (Figure 6c,g). By performing MSOT signal averaging along both lateral coordinates, depth profiles of the corresponding chromophores were obtained (Figure 6e). The mean relative error of approximation was <10%. Areas with the highest absorption of each component are highlighted in Figure 6f,g with the corresponding spectra perfectly fitting those of water (Figure 6f) and lipids (Figure 6g).

Most importantly, MSOT enables clearly identifying the muscle layer after performing signal averaging below the hypodermis layer (area marked with “h” in Figure 6c), revealing a clear characteristic local maximum of collagen around the 910 nm wavelength (Figure 5h).

Hence, spectral unmixing of the MSOT data allowed direct imaging of three skin layers: the dermis, characterized by the dominant water absorption peak (region “f” in Figure 6b and its corresponding spectrum in Figure 6f), the hypodermis, characterized by the absorption of lipids (region “g” in Figure 6c and its corresponding spectrum in Figure 6g), and the muscles layer below the hypodermis, where the average spectrum exhibits the collagen‐related peak at 910 nm (region “h” in Figure 6c and its corresponding spectrum in Figure 6h).

## 3 Discussion

Collagen is a major structural protein in the extracellular matrix of muscles and plays a crucial role in maintaining muscle integrity, elasticity, and function.^[^ ^52^ ^]^ Changes in collagen content and organization can indicate various physiological and pathological conditions, including aging‐related muscle degeneration, fibrosis, and sarcopenia.^[^ ^1^ ^]^ It has been demonstrated that the different shapes of vibrational spectra of proteins, lipids, and water determine the different positions of the absorption maxima of the overtones of these molecules' vibrations. For instance, the absorption band of water in the region of 970 nm is determined by the overtone of O‐H vibrations, which arises as the sum a *a* *v*_1_ +  *b* *v*_3_;  *a*  +  *b*  =  3, *v*_1_ and *v*_2_ are the frequencies of symmetric and antisymmetric vibrations, respectively.^[^ ^53^ ^]^ Similarly, the difference in the frequencies of C─H vibrations in proteins, amino acids, and lipids leads to differences in their NIR absorption spectra. This can potentially be used for diagnostic purposes^[^ ^13^, ^26^, ^39^, ^41^, ^54^, ^55^ ^]^ and spectral unmixing, e.g. assessment of collagen is facilitated by its characteristic maximum ≈ 910 nm in absorption and diffuse reflectance spectra.^[^ ^56^ ^]^

Here we demonstrated that, beyond the hemoglobin components, three other intrinsic tissue constituents, namely, collagen, lipid, and water, can effectively be extracted via noninvasive DRS and MSOT measurements in the NIR spectrum. Importantly, higher density of the CH‐oscillators per molecule makes lipid absorption several times more prominent compared to collagen for the same mass concentration (Figure 2c,d and Table 2), thus challenging its sensitive assessment in lipid rich tissues. The amplitude of the C─H absorption band for collagen (near the local maximum at 910 nm) is three times lower than that of the C─H absorption band for lipids (near the local maximum at 930 nm). In addition, the proteins (including collagen) make up only ≈ 20% of the dermis and muscle layers, whereas lipid content in the adipose layer exceeds 80%,^[^ ^57^, ^58^ ^]^ resulting in an additional ratio of at least 4:1 by mass. At the same mass fraction, the number of C─H bonds in lipids is approximately three times higher compared to proteins (Table 2). Therefore, C─H absorption band at 930 nm exceeds by at least order of magnitude the collagen absorption peak at 910 nm. Since DRS measures an integrated signal along the entire diffuse photon propagation path, the muscle signal is overshadowed in the presence of a thick adipose layer.

Nevertheless, in subjects with thin hypodermis and skin areas free of pronounced hypodermis (e.g., palm), the collagen‐related peak can be identified in the DRS data at source‐detector separation >5 mm (Figure 3). The fact that the protein‐related peak increases with the probing depth indicates that it originates from the deeper muscle layer rather than from the upper dermis layer. Moreover, we observed a clear correlation between the amplitude of the collagen peak in the optical density spectra and muscles percentage in the body as measured by mBIA (Figure 4). Hence, this spectral indicator can in principle be used for muscle assessments. Yet, in the presence of a pronounced hypodermis, the peak of lipids at 930 nm completely masks the protein peak at 910 nm, making quantifications impossible. The study was conducted on a cohort of 72 subjects, which implies some variability in both skin phototype and upper skin layer thickness. Skin phototype is primarily determined by the melanin content of the basal layer of the epidermis. As this layer is much thinner than the dermis and hypodermis while no pronounced melanin absorption peaks exist in the examined spectral range, we assume that phototype has a negligible effect on the recorded signal. In contrast, skin thickness varies considerably between anatomical locations, hence the prediction models should be recalibrated with newly acquired paired DRS spectra and reference measurements before applying the technique to another body region.

In contrast, MSOT can selectively detect the collagen signal originating from muscles due to the high spatial resolution of the method along the depth direction. Using spectral unmixing, we successfully identified and localized three spectrally distinct tissue layers (dermis, hypodermis and muscles) and experimentally retrieved their averaged spectra (Figure 5f–h). Hence, the high spatial precision of MSOT allows isolating the contributions from weakly absorbing collagen proteins in the presence of the relatively high absorption by lipids. This technique therefore allows for the assessment of collagen in muscles not accessible by DRS.

It should be noted that the widespread adoption of MSOT is hampered by the relative complexity of its experimental setup and the time required for data acquisition and signal processing. In contrast, the inherently simple hardware implementation of DRS—and the possibility of reducing it to a system comprising only light‑emitting diodes and photodiodes—opens the door to a wearable device for rapid, point‑of‑care body‑composition assessment.

Thus, the two modalities represent a functionally complementary unit with the DRS method making it possible to quantify water and lipid content in the skin and collagen content in muscle when the hypodermis is absent at the selected body site (e.g., the palm or, in some individuals, the forearm). Due to its higher spatial resolution, MSOT can additionally resolve the depth‐dependent distribution of water, lipids and collagen, which is potentially useful for subcutaneous fat‐rich regions. However, potential spectral overlap between lipid and collagen absorption peaks can still pose a challenge to signal interpretation in regions where these chromophores are not spatially separated.

A promising direction for future research is the integration of DRS and MSOT into a single instrument, further complemented by ultrasound imaging to enable comprehensive assessment of tissue structure and composition. This can be achieved using multi‐element piezopolymer ultrasound arrays, which allow for simultaneous detection of optical illumination,^[^ ^59^ ^]^ realization of optical tomography,^[^ ^60^ ^]^ and acquisition of ultrasound, optical, and optoacoustic data.^[^ ^61^ ^]^ The development of such a multimodal system would significantly enhance the non‐invasive characterization of biological tissues.

## 4 Conclusion

In this study, we highlight the potential of advanced optical spectroscopy techniques, namely, DRS and MSOT, for the non‐invasive analysis of collagen and other tissue components in the human skin and subcutaneous tissues. Our findings underscore the significance of collagen as a marker for muscle tissue, particularly in areas with thinner hypodermis, providing new insights into tissue composition and health assessment. The ability to detect collagen absorption more prominently with DRS at larger source‐detector separations is a promising new finding, suggesting a strong association with underlying muscle structures. Furthermore, the multi‐modal integration between the DRS and MSOT methods offers promising avenues for future diagnostic applications employing multi‐parametric characterization of tissue components.

## 5 Experimental Section

### Protocol for Gelatin Phantoms Fabrication

The phantoms were fabricated according to the following protocol. Gelatin powder was dissolved in distilled water. A solution of TiO_2_ powder was used as a scatterer, so that the powder concentration in the phantom was 0.6 g L^−1^. To achieve a uniform concentration of gelatin and TiO_2_ powder in the phantom, the mixture was stirred in a water bath at 60 °C. Additionally, degassing of the phantom was performed in a vacuum chamber to remove air bubbles from the phantom volume.

In a series of experiments on model structures, three gelatin phantoms with a concentration of 100 g L^−1^ were prepared, having thicknesses of 2, 3, and 4 mm. A gelatin phantom with nigrosine dye was used as the second layer (substrate). Nigrosine was added to the substrate phantom to reduce the back‐reflection of the generated optoacoustic signal from the bottom surface of the sample. These samples were made to investigate the possibility of determining thickness of the first layer in multi‐layered phantoms using MSOT.

In the first layer, water was assumed to be the main absorber. This allowed to model optical properties of the dermis. The second layer represented the remaining subcutaneous structures – the hypodermis and muscles. Consequently, the proposed two‐layer model primarily describes the dermis. As the thickness of epidermis does not exceed 100 µm,^[^ ^41^ ^]^ which is far below the spatial resolution of the DRS and MSOT techniques used, its contribution was neglected. The absence of a detailed consideration of the optical properties of hypodermis and muscles represents a limitation of the model.

### Absorption Spectra of Collagen, Lipid, and Water

To record the absorption spectra of the overtone vibrational modes of collagen, lipid, and water, measurements were performed for solutions of albumin, l‐alanine, and gelatin dissolved in heavy water using a Lambda‐25 (Perkin‐Elmer, USA) spectrophotometer in the range of 700–1100 nm. In this way, this could measure the molar extinction spectra of collagen and lipid molecules. The concentrations of substances are listed in **Table** **3**. The absorption spectra of distilled water and lipids are known from the literature.^[^ ^58^, ^62^ ^]^ In addition, for each type of molecule, the specific molar extinction coefficient normalized to the number of C─H bonds per molecule was calculated. The number of C─H bonds per molecule, as well as their molar masses, was determined using an open‐access database.^[^ ^49^ ^]^ Since special attention was paid in this study to the formation of the optical spectra in the NIR range by molecules that have C─H bonds, the absorption spectra of pentadecane were also considered. This was done to evaluate the feasibility of determining the protein and lipid content in skin by analyzing absorption spectra in the 700–1100 nm range.

**Table 3:** Concentrations of substances dissolved in heavy water used for measuring the Raman and absorption spectra.

№	Sample	Concentrations [gL^−1^]
1	Albumin	100
2	L‐alanine	100
3	Collagen	200

### Raman Spectra of Collagen, Lipid, and Water

To measure the vibrational spectra of collagen, lipid, and water, Raman spectra were obtained for ex vivo fat sample, distilled water, solutions of albumin and l‐alanine in heavy water under laser excitation at a wavelength of 690 nm using High Performance Spectrometer with TE‐Cooling YIXIST (China; spectral resolution 2.3 nm). The laser power on the sample was 25 mW and the time acquisition of Raman signal was 3 s. Concentrations of substances in the solutions are listed in Table 3.

### Diffuse Reflectance and MSOT Spectra of Collagen, Lipids, and Water

To investigate the contribution of collagen, lipid, and water to the diffuse reflectance signal, DRS and MSOT spectral measurements of ex vivo fat samples were conducted, and gelatin phantoms based on a regular as well as heavy water.

### In Vivo Measurements of Skin Using DRS and MSOT

The experiment involved a sample of 72 volunteers (19 males, 53 females). Using an experimental setup for DRS and MSOT, measurements on different areas of the skin on the arm were conducted. The following skin areas were considered: palm, forearm, and biceps. Skin areas were selected without obvious damage and with minimal hair coverage. The analyzed subjects comprised volunteers with skin phototypes ranging from I to IV. Informed consent was obtained from all participants. The studies involving human participants were reviewed and approved by the Local Ethics Committee of the Medical Research and Educational Institute, Lomonosov Moscow State University (code of protocol 8; date of protocol:13.10.2023).

### Measurement and Preprocessing of Diffuse Reflectance Spectra

An experimental setup was used for measuring diffuse reflectance spectra as described in previous works.^[^ ^15^, ^50^ ^]^ Briefly, it allows measuring diffuse reflectance spectra in the spectral range of 800–1100 nm using a fiber optic probe. The distance between the source and detector fibers varied from 0 to 15 mm. This variation allows for spatially resolved measurements of diffuse reflectance spectra.

The effective optical density was calculated using the following Equation (1):

(1) $$\mathrm{OD}\left(\lambda \right)=-\log_{10}\left(\left(I_{\mathrm{skin}}\left(\lambda \right)-I_{\mathrm{dark}}\left(\lambda \right)\right)/\left(I_{\mathrm{ref}}\left(\lambda \right)-I_{\mathrm{dark}}\left(\lambda \right)\right)\right)$$

Here, *I_skin_* (λ) is the reflected spectrum from the skin, *I_dark_* (λ) is the dark noise of the spectrometer, *I_ref_* (λ) is the reflected spectrum from the standard.

The collagen content (Collagen Index) was evaluated by subtracting a line intersecting the spectrum at 900 and 915 nm. The average value of the effective optical density in this spectral range was then calculated.

The water content (Water Index, WI) and lipid content (Lipids Index, LI) were evaluated according to the specified formulas:

(2) $$\mathrm{WI}=\mathrm{OD}\left(970\mathrm{nm}\right)-\mathrm{OD}\left(850\mathrm{nm}\right)$$

(3) $$LI=OD\left(930\;nm\right)-OD\left(850\;nm\right)$$

The study sample was divided into two subgroups. The first subgroup (n_1_ = 61) consisted of volunteers with a visually assessed thin hypodermis, for whom no lipid absorption peak around 930 nm was observed in the diffuse reflectance spectra measured at a source‐detector distance of 15 mm. The second subgroup (n_2_ = 11) consisted of volunteers with a thick hypodermis, for whom a lipid absorption peak near 930 nm could be detected in the diffuse reflectance spectra.

### Multispectral Optoacoustic Tomography (MSOT) Setup

The system for volumetric MSOT imaging was based on a broadband hand‐held custom 256‐element spherical matrix array transducer (BARI‐NN Ltd., Russia) with 40 mm focal distance and 90 degrees angular coverage. The individual array elements have a 3 mm diameter, 0.1‐20 MHz detection bandwidth, and <10 Pa noise equivalent pressure. The optical pulses with repetition rate of 20 Hz were delivered through 8 mm antenna central hole from a tunable nanosecond laser (Model: Photosonus, Ekspla, Lithuania) using a fiber bundle (CeramOptec, Germany) with 0.22 numerical aperture. Signals detected by the array were digitized at 40 Mega samples per second by multi‐channel electronics (Model: Legion, Photosound, USA) via custom computer interface in the Matlab environment. Acoustic and optical coupling between the array and the sample surface were provided by an immersion chamber filled with heavy water (D_2_O).

Optoacoustic measurements were carried out in the wavelength range from 800 to 1100 nm with 10 nm steps. The measured data was normalized to the laser energy for each excitation wavelength. Since collecting the MSOT dataset required ≈ 3 s and was affected by motion artifacts due to cardiac activity, a low‐pass filter with a cutoff frequency of 1.5 MHz was applied to every A‐scan. Each volumetric dataset was processed by a back‐projection reconstruction algorithm to render 10×10×10 mm^3^ volume with 100 µm voxels.^[^ ^63^ ^]^ The MSOT signal was then spectrally unmixed on a voxel‐by‐voxel basis to identify components related to the concentrations of water, lipids, and other absorbers. For this, fitting of the MSOT spectra was performed using the Equation (3):

(4) $$\begin{array}{ccc} & & p_{proc}\;\left(r,\lambda \right)\\ & & \;=\left(c_{water}\left(r\right)\mu_{a}^{water}\left(\lambda \right)+c_{lipids}\left(r\right)\mu_{a}^{lipids}\left(\lambda \right)+c_{0}\left(r\right)+c_{1}\left(r\right)\lambda +c_{2}\left(r\right)\lambda^{2}\right) \end{array}$$

where $\mu_{a}^{water}\left(\lambda \right)$  is the absorption spectrum of water, $\mu_{a}^{lipids}\left(\lambda \right)$  is the absorption spectrum of lipids. In the case of phantom measurements, the second‐degree polynomial term accounts for the contribution of nigrosine absorption. In the case of in vivo skin measurements, this term accounts for hemoglobin absorption, as no characteristic local maxima for these components are observed in the spectral range used.

### Multispectral Bioimpedance Analysis (mBIA)

Body composition (fat, water, lean mass and muscle mass) was measured using the InBody 770 device (Biospace Co., USA), which uses mBIA to assess body composition. Measurements using the device were carried out according to the manufacturer's recommendations. Weight was estimated using this device; height and gender were indicated in the device as part of the measurement and height and weight were used to calculate the participant's BMI.

### Statistical Analysis

T‐test for independent samples was used for comparing mBIA parameters between groups with thick and thin hypodermis (n_1_ = 62, n_2_ = 11). It wass estimated that, at the given significance level of 0.01, the effect size (difference between mean values in sample to standard deviation) of 0.8 is detected with 50% sensitivity, effect size of 1.29 with 90% sensitivity, and effect size of 1.65 with 99% sensitivity. In addition to the Pearson's correlation between different parameters reconstructed using DRS, the test power was estimated for sample used in experiments (n = 72). The used number of objects in the sample allows to avoid type II errors (false negatives in the presence of correlation) with sensitivity of 50% at r = 0.3 at a given significance level of 0.01, 83% at r = 0.44, and >99% at r > 0.53, i.e., even weak correlations at the level of r = 0.3‐0.4 should be detected in such a sample. All calculations were performed with the SciPy, statsmodels libraries in Python.

## Conflict of Interest

The authors declare no conflict of interest.

## Supporting information



Supporting Information

## Data Availability

The data that support the findings of this study are available from the corresponding author upon reasonable request.

## References

[1] S. Yuan , S. C. Larsson , Metabolism 2023, 144, 155533. 36907247 10.1016/j.metabol.2023.155533

[2] J.‐G. Wang , Y. Zhang , H.‐E. Chen , Y. Li , X.‐G. Cheng , L. Xu , Z. Guo , X.‐S. Zhao , T. Sato , Q.‐Y. Cao , K.‐M. Chen , B. Li , The J. Strength Condition. Res. 2013, 27, 236. 10.1519/JSC.0b013e31824f2040 22344056

[3] C. Eklouh‐Molinier , T. Happillon , N. Bouland , C. Fichel , M.‐D. Diébold , J.‐F. Angiboust , M. Manfait , S. Brassart‐Pasco , O. Piot , Analyst 2015, 140, 6260. 26120602 10.1039/c5an00278h

[4] O. A. Smolyanskaya , N. V. Chernomyrdin , A. A. Konovko , K. I. Zaytsev , I. A. Ozheredov , O. P. Cherkasova , M. M. Nazarov , J.‐P. Guillet , S. A. Kozlov , Y. V. Kistenev , J.‐L. Coutaz , P. Mounaix , V. L. Vaks , J.‐H. Son , H. Cheon , V. P. Wallace , Y. Feldman , I. Popov , A. N. Yaroslavsky , A. P. Shkurinov , V. V. Tuchin , Quantum Electron 2018, 62, 1.

[5] S. S. Gazzotti Rebecca , A. G. M. Pilar , G. Riccardo , V. N. Violeta , M. Carmelo , G. Giuseppe , B. Alberto , I. B. Composition , Semin. Musculoskelet Radiol. 2024, 28, 594.39406222

[6] J. A. Shepherd , B. K. Ng , M. J. Sommer , S. B. Heymsfield , Bone 2017, 104, 101. 28625918 10.1016/j.bone.2017.06.010 PMC5659281

[7] A. Różdżyńska‐Świątkowska , E. Jurkiewicz , A. Tylki‐Szymańska , in JIMD Reports, (Eds.: E. Morava , M. Baumgartner , M. Patterson , S. Rahman , J. Zschocke , V. Peters ), Springer, Berlin Heidelberg, Berlin, Heidelberg 2016, 26, pp. 45. 10.1007/8904_2015_473 PMC4864712 26253708

[8] S. Lv , L. Ling , H. Shi , X. Chen , S. Chen , S. Zhu , W. Lin , R. Lv , G. Ding , Front. Med. (Lausanne) 2022, 9, 859555. 35433721 10.3389/fmed.2022.859555 PMC9009442

[9] M. A. Islam , K. Sundaraj , R. B. Ahmad , N. U. Ahamed , PLoS One 2013, 8, 58902. 10.1371/journal.pone.0058902 PMC3594217 23536834

[10] J. S. Nyman , A. J. Makowski , C. A. Patil , T. P. Masui , E. C. O'Quinn , X. Bi , S. A. Guelcher , D. P. Nicollela , A. Mahadevan‐Jansen , Calcif. Tissue Int. 2011, 89, 111. 21597909 10.1007/s00223-011-9497-x PMC4471954

[11] B. P. Yakimov , E. A. Shirshin , J. Schleusener , A. S. Allenova , V. V. Fadeev , M. E. Darvin , Sci. Rep. 2020, 10, 14374. 32873804 10.1038/s41598-020-71220-6 PMC7463016

[12] X. Chen , O. Nadiarynkh , S. Plotnikov , P. J. Campagnola , Nat. Protoc. 2012, 7, 654. 22402635 10.1038/nprot.2012.009 PMC4337962

[13] S.‐Y. Tzeng , T.‐Y. Kuo , S.‐B. Hu , Y.‐W. Chen , Y.‐L. Lin , K.‐Y. Chu , S.‐H. Tseng , Skin Res. Technol. 2018, 24, 59. 28771835 10.1111/srt.12390

[14] E. Shirshin , B. Yakimov , D. Davydov , A. Baev , G. Budylin , N. Fadeev , L. Urusova , N. Pachuashvili , O. Vasyukova , N. Mokrysheva , Anal. Methods 2024, 16, 175. 38099917 10.1039/d3ay01901b

[15] D. A. Davydov , G. S. Budylin , A. V. Baev , D. V. Vaypan , E. M. Seredenina , S. T. Matskeplishvili , S. A. Evlashin , A. A. Kamalov , E. A. Shirshin , J. Biomed. Opt. 2023, 28, 057002. 37193365 10.1117/1.JBO.28.5.057002 PMC10182858

[16] G. S. Budylin , D. A. Davydov , N. V. Zlobina , A. V. Baev , V. G. Artyushenko , B. P. Yakimov , E. A. Shirshin , J. Biophotonics 2022, 15, 202100268. 10.1002/jbio.202100268 34661967

[17] I. Ivankovic , H.‐C. A. Lin , A. Özbek , A. Orive , X. L. Deán‐Ben , D. Razansky , Adv. Sci. 2024, 11, 2308336. 10.1002/advs.202308336 PMC11095142 38445972

[18] X. L. Deán‐Ben , S. Gottschalk , B. Mc Larney , S. Shoham , D. Razansky , Chem. Soc. Rev. 2017, 46, 2158. 28276544 10.1039/c6cs00765a PMC5460636

[19] V. Perekatova , A. Kostyuk , M. Kirillin , E. Sergeeva , D. Kurakina , O. Shemagina , A. Orlova , A. Khilov , I. Turchin , Diagnostics 2023, 13, 457. 36766562 10.3390/diagnostics13030457 PMC9913927

[20] I. Turchin , V. Beschastnov , P. Peretyagin , V. Perekatova , A. Kostyuk , A. Orlova , N. Koloshein , A. Khilov , E. Sergeeva , M. Kirillin , Biomedicines 2023, 11, 351. 36830888 10.3390/biomedicines11020351 PMC9953239

[21] B. P. Yakimov , D. A. Davydov , V. V. Fadeev , G. S. Budylin , E. A. Shirshin , Quantum Elec (Woodbury) 2020, 50, 41.

[22] D. А. Davydov , G. S. Budylin , A. V. Baev , D. V. Vaipan , E. M. Seredenina , A. A. Kamalov , E. A. Shirshin , J. Biophotonics 2024, 18, 202300509. 10.1002/jbio.202300509 38185913

[23] R. V. Warren , R. Bar‐Yoseph , B. Hill , D. Reilly , A. Chiu , S. Radom‐Aizik , D. M. Cooper , B. J. Tromberg , J. Biomed. Opt. 2022, 27, 065002. 35676754 10.1117/1.JBO.27.6.065002 PMC9176379

[24] I. Bardadin , V. Petrov , G. Denisenko , A. Armaganov , A. Rubekina , D. Kopytina , V. Panov , P. Shatalov , V. Khoronenko , P. Shegai , Photonics 2024, 11, 49.

[25] B. P. Yakimov , K. E. Buiankin , A. V. Venets , E. A. Shirshin , J. Biomed. Photon. Eng. 2022, 8, 040509.

[26] S.‐H. Tseng , S.‐Y. Tzeng , Y.‐K. Liaw , C.‐K. Hsu , J. Lee , W.‐R. Chen , J. Biomed. Opt. 2012, 17, 0770051. 10.1117/1.JBO.17.7.077005 22894518

[27] N. Serra , R. Cubeddu , G. Maffeis , V. Damagatla , A. Pifferi , P. Taroni , Sci. Rep. 2024, 14, 19154. 39160254 10.1038/s41598-024-70099-x PMC11333589

[28] D. A. Nazarov , G. M. Denisenko , G. S. Budylin , E. A. Kozlova , M. M. Lipina , V. A. Lazarev , E. A. Shirshin , M. K. Tarabrin , Biomed. Opt. Express 2023, 14, 1509. 37078039 10.1364/BOE.483135 PMC10110295

[29] X. Luís Deán‐Ben , D. Razansky , Light Sci Appl. 2014, 3, 137. 10.1038/lsa.2018.4 PMC6060052 30839533

[30] J. Rebling , M. Ben‐Yehuda Greenwald , M. Wietecha , S. Werner , D. Razansky , Adv. Sci. 2021, 8, 2004226. 10.1002/advs.202004226 PMC8261523 34258153

[31] A. Korobov , Z. Besedovskaia , E. Petrova , A. Kurnikov , A. Glyavina , A. Orlova , S. Nemirova , I. Druzhkova , M. Sirotkina , E. Shirshin , D. Gorin , L. Xi , D. Razansky , P. Subochev , J. Biophotonics 2024, 17, 202400143. 10.1002/jbio.202400143 39384323

[32] S. A. Ermilov , T. Khamapirad , A. Conjusteau , M. H. Leonard , R. Lacewell , K. Mehta , T. Miller , A. A. Oraevsky , J. Biomed. Opt. 2009, 14, 024007. 19405737 10.1117/1.3086616

[33] P. V. Subochev , X. L. Deán‐Ben , Z. Chen , M. B. Prudnikov , V. A. Vorobev , A. A. Kurnikov , A. G. Orlova , A. S. Postnikova , A. V. Kharitonov , M. D. Proyavin , R. I. Ovsyannikov , A. G. Sanin , M. Y. Kirillin , F. Montero de Espinosa , I. V. Turchin , D. Razansky , Light Sci. Appl. 2025, 14, 239. 40623976 10.1038/s41377-025-01894-y PMC12234755

[34] S. K. Kalva , A. Sánchez‐Iglesias , X. L. Deán‐Ben , L. M. Liz‐Marzán , D. Razansky , ACS Appl. Mater. Interfaces 2022, 14, 172. 34949083 10.1021/acsami.1c17661

[35] E. Merčep , X. L. Deán‐Ben , D. Razansky , IEEE Trans. Med. Imaging 2017, 36, 2129. 28541198 10.1109/TMI.2017.2706200

[36] A. L. Wagner , V. Danko , A. Federle , D. Klett , D. Simon , R. Heiss , J. Jüngert , M. Uder , G. Schett , M. F. Neurath , J. Woelfle , M. J. Waldner , R. Trollmann , A. P. Regensburger , F. Knieling , Photoacoustics 2021, 21, 100220. 33318928 10.1016/j.pacs.2020.100220 PMC7723806

[37] A. Karlas , N.‐A. Fasoula , N. Katsouli , M. Kallmayer , S. Sieber , S. Schmidt , E. Liapis , M. Halle , H.‐H. Eckstein , V. Ntziachristos , Photoacoustics 2023, 30, 100468. 36950518 10.1016/j.pacs.2023.100468 PMC10025091

[38] X. L. Deán‐Ben , D. Razansky , Front. Phys. 2022, 10, 1028258.

[39] A. P. Regensburger , L. M. Fonteyne , J. Jüngert , A. L. Wagner , T. Gerhalter , A. M. Nagel , R. Heiss , F. Flenkenthaler , M. Qurashi , M. F. Neurath , N. Klymiuk , E. Kemter , T. Fröhlich , M. Uder , J. Woelfle , W. Rascher , R. Trollmann , E. Wolf , M. J. Waldner , F. Knieling , Nat. Med. 2019, 25, 1905. 31792454 10.1038/s41591-019-0669-y

[40] K. Tascilar , F. Fagni , A. Kleyer , S. Bayat , R. Heidemann , F. Steiger , G. Krönke , D. Bohr , A. Ramming , F. Hartmann , D. Klett , A. Federle , A. P. Regensburger , A. L. Wagner , F. Knieling , M. F. Neurath , G. Schett , M. Waldner , D. Simon , Rheumatology 2023, 62, 841. 35699479 10.1093/rheumatology/keac346

[41] D. Jüstel , H. Irl , F. Hinterwimmer , C. Dehner , W. Simson , N. Navab , G. Schneider , V. Ntziachristos , Adv. Sci. 2023, 10, 2301322. 10.1002/advs.202301322 PMC10323611 37092572

[42] Z. Chen , I. Gezginer , Q. Zhou , L. Tang , X. L. Deán‐Ben , D. Razansky , Chem. Soc. Rev. 2024, 53, 6068. 38738633 10.1039/d3cs00565h PMC11181994

[43] A. Q. Bauer , R. E. Nothdurft , J. P. Culver , T. N. Erpelding , L. V. Wang , J. Biomed. Opt. 2011, 16, 096016. 21950930 10.1117/1.3626212 PMC3188642

[44] Y. Wang , J. Li , T. Lu , L. Zhang , Z. Zhou , H. Zhao , F. Gao , Appl. Opt. 2017, 56, 303. 28085867 10.1364/AO.56.000303

[45] L. Xi , X. Li , L. Yao , S. Grobmyer , H. Jiang , Med. Phys. 2012, 39, 2584. 22559629 10.1118/1.3703598

[46] P. D. Kumavor , C. Xu , A. Aguirre , J. K. Gamelin , Y. Ardeshirpour , B. Tavakoli , S. Zanganeh , U. S. Alqasemi , Y. Yang , Q. Zhu , J. Biomed. Opt. 2011, 16, 046010. 21529079 10.1117/1.3563534 PMC3087425

[47] K. G. Akhmedzhanova , A. A. Kurnikov , D. A. Khochenkov , Y.u. A. Khochenkova , A. M. Glyavina , V. V. Kazakov , A. V. Yudintsev , A. V. Maslennikova , I. V. Turchin , P. V. Subochev , A. G. Orlova , Biomed. Opt. Express 2022, 13, 5695. 36733761 10.1364/BOE.469380 PMC9872889

[48] A. Orlova , K. Pavlova , A. Kurnikov , A. Maslennikova , M. Myagcheva , E. Zakharov , D. Skamnitskiy , V. Perekatova , A. Khilov , A. Kovalchuk , A. Moiseev , I. Turchin , D. Razansky , P. Subochev , Neoplasia 2022, 26, 100778. 35220045 10.1016/j.neo.2022.100778 PMC8889238

[49] “National Center for Biotechnology Information ,” https://www.ncbi.nlm.nih.gov/, (accessed: July 2025)

[50] D. A. Davydov , N. A. Fadeev , I. D. Filippov , G. S. Budylin , J. Biomed. Photonics Eng. 2024, 10, 21.

[51] D. Geraskin , H. Boeth , M. Kohl‐Bareis , J. Biomed. Opt. 2009, 14, 044017. 19725728 10.1117/1.3184425

[52] M. D. Shoulders , R. T. Raines , Annu. Rev. Biochem. 2009, 78, 929. 19344236 10.1146/annurev.biochem.77.032207.120833 PMC2846778

[53] G. M. Hale , M. R. Querry , Appl. Opt. 1973, 12, 555. 20125343 10.1364/AO.12.000555

[54] P. Taroni , A. M. Paganoni , F. Ieva , A. Pifferi , G. Quarto , F. Abbate , E. Cassano , R. Cubeddu , Sci. Rep. 2017, 7, 40683. 28091596 10.1038/srep40683 PMC5238417

[55] M. Peyvasteh , A. Popov , A. Bykov , I. Meglinski , J. Phys. Commun. 2020, 4, 095011.

[56] S. K. V. Sekar , I. Bargigia , A. D. Mora , P. Taroni , A. Ruggeri , A. Tosi , A. Pifferi , A. Farina , J. Biomed. Opt. 2017, 22, 015006. 10.1117/1.JBO.22.1.015006 28138693

[57] A. N. Bashkatov , E. A. Genina , V. V. Tuchin , J. Innov. Opt. Health Sci. 2011, 04, 9.

[58] S. L. Jacques , Phys. Med. Biol. 2013, 58, R37. 23666068 10.1088/0031-9155/58/11/R37

[59] P. Subochev , I. Fiks , M. Frenz , Turchin , Laser Phys. Lett. 2016, 13, 025605.

[60] P. Subochev , Opt. Lett. 2016, 41, 1006. 26974102 10.1364/OL.41.001006

[61] P. Subochev , A. Orlova , I. Mikhailova , N. Shilyagina , I. Turchin , Biomed. Opt. Express 2016, 7, 3951. 27867706 10.1364/BOE.7.003951 PMC5102546

[62] R. L. P. Van Veen , H. Sterenborg , A. Pifferi , A. Torricelli , R. Cubeddu , in Proc. of Biomedical Topical Meetings , Citeseer, New Jersey 2000.

[63] X. L. Deán‐Ben , A. Ozbek , D. Razansky , IEEE Trans. Med. Imaging 2013, 32, 2050. 23846468 10.1109/TMI.2013.2272079

