# Supplementary material for: Water, Collagen, and Lipid Content in the Human Skin and Muscles Assessed with Near‐Infrared Diffuse Reflectance Spectroscopy and Multi‐Spectral Optoacoustic Tomography
Source: Adv Sci (Weinh). 2025 Aug 13;12(41):e05619. doi: 10.1002/advs.202505619 (PMC12591192; doi:10.1002/advs.202505619)
Supplement: Supplementary file 1 — Supporting Information [file ADVS-12-e05619-s001.pdf]

Supporting Information

Water, collagen and lipid content in the human skin and muscles assessed with NIR multispectral optoacoustic tomography and diffuse reflectance spectroscopy

Denis Davydov, Alexey Kurnikov, Pavel Subochev, Gleb Budylin, Nikolay Fadeev, Ivan Filippov, Natalia Mokrysheva, Liliya Urusova, Daniel Razansky, Evgeny Shirshin**

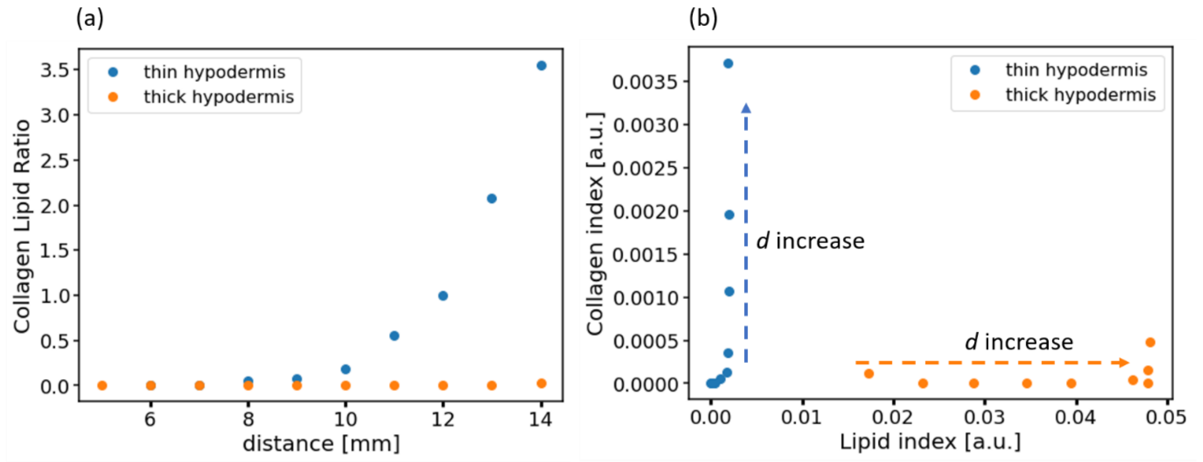

Figure S1. (a) Dependence of ratio Collagen Index (absorption at 910 nm) to Lipid Index (absorption at 930 nm) on source-detector separation for subjects with thin and thick hypodermis. (b) Dependence of DRS-derived Collagen Index (absorption at 910 nm) and Lipid Index (absorption at 930 nm) on source-detector separation for subjects with thin and thick hypodermis. Each point corresponds to a specific source-detector separation.

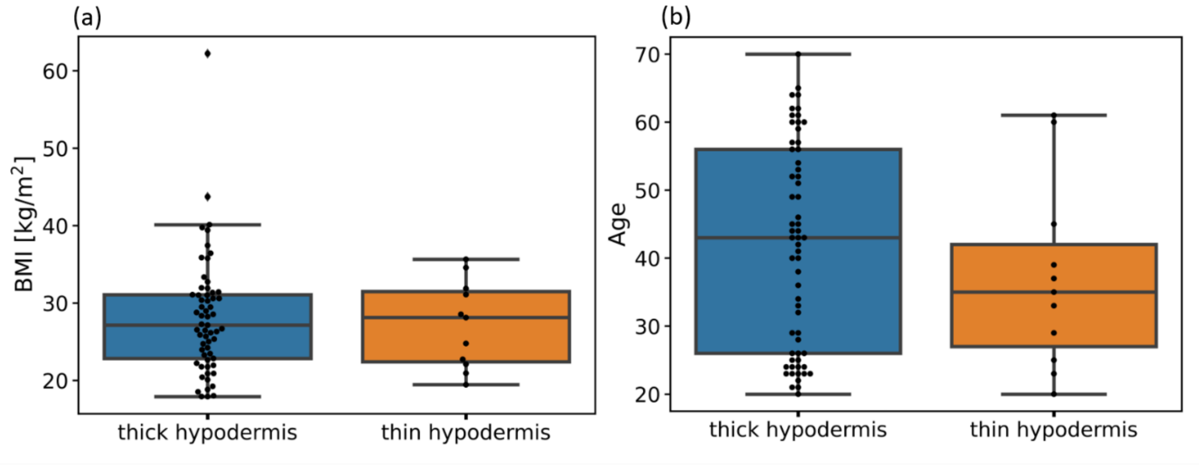

Figure S2. (a) Box plots of the body mass index (BMI) for volunteers with thick and thin hypodermis. (b) Box plots of the age for volunteers with thick and thin hypodermis.
